# Screening Genes Promoting Exit from Naive Pluripotency Based on Genome-Scale CRISPR-Cas9 Knockout

**DOI:** 10.1155/2020/8483035

**Published:** 2020-02-03

**Authors:** Bin Yang, Junqi Kuang, Chuman Wu, Wenyi Zhou, Shuoji Zhu, Haodong Jiang, Ziwei Zhai, Yue Wu, Junwei Peng, Nanbo Liu, Haiyan Hu, Nasser Moussa Ide, Ruiping Chen, Mingyi Zhao, Ping Zhu

**Affiliations:** ^1^The Second School of Clinical Medicine, Southern Medical University, Guangzhou, Guangdong 510515, China; ^2^Guangdong Institute of Cardiovascular Disease, Guangdong Provincial People's Hospital, Guangdong Academy of Medical Sciences, Guangzhou, Guangdong 510080, China; ^3^Guangzhou Institutes of Biomedicine and Health, Chinese Academy of Sciences, Guangzhou, Guangdong 510530, China; ^4^School of Medicine, South China University of Technology, Guangzhou, Guangdong 510006, China; ^5^Department of Cardiothoracic Surgery of East Division, The First Affiliated Hospital, Sun Yat-sen University, Guangzhou, Guangdong 510080, China; ^6^Department of Pediatrics, The Third Xiangya Hospital, Central South University, Changsha, Hunan 410013, China

## Abstract

Two of the main problems of stem cell and regenerative medicine are the exit of pluripotency and differentiation to functional cells or tissues. The answer to these two problems holds great value in the clinical translation of stem cell as well as regenerative medicine research. Although piling researches have revealed the truth about pluripotency maintenance, the mechanisms underlying pluripotent cell self-renewal, proliferation, and differentiation into specific cell lineages or tissues are yet to be defined. To this end, we took full advantage of a novel technology, namely, the genome-scale CRISPR-Cas9 knockout (GeCKO). As an effective way of introducing targeted loss-of-function mutations at specific sites in the genome, GeCKO is able to screen in an unbiased manner for key genes that promote exit from pluripotency in mouse embryonic stem cells (mESCs) for the first time. In this study, we successfully established a model based on GeCKO to screen the key genes in pluripotency withdrawal. Our strategies included lentiviral package and infection technology, lenti-Cas9 gene knockout technology, shRNA gene knockdown technology, next-generation sequencing, model-based analysis of genome-scale CRISPR-Cas9 knockout (MAGeCK analysis), GO analysis, and other methods. Our findings provide a novel approach for large-scale screening of genes involved in pluripotency exit and offer an entry point for cell fate regulation research.

## 1. Introduction

As a type of undifferentiated primitive cells with the potential of self-renewal and multilineage differentiation, pluripotent stem cells could differentiate into any kind of cell type and then further develop into any tissue or organ. Pluripotency is governed by a transcription factor network that contains numerous autoregulatory loops. And the exit from pluripotency means the end of this very recursive circuitry to enable developmental progression and lineage commitment, which is of high application value in the fields of regenerative medicine, disease model, drug screen, cell fate regulation, and ontogenesis. Currently, numerous studies focus on the maintenance of pluripotency, while few look into the regulatory machinery in pluripotency exit.

There are numerous factors that help maintain pluripotency. However, the mechanism by which the cells exit from the proliferation cycle orderly, activate the development process, and eventually differentiate into specific lineage is yet to be defined. Revealing the mechanisms underlying pluripotency exit may contribute to the finding of critical points that control pluripotency. To balance the proliferation and differentiation, the levels of transcriptional factors, including *Oct4*, *Sox2*, and *Nanog*, must be well controlled. These genes play a vital role in maintaining pluripotency.

Meanwhile, certain transcriptional factors are also needed to prevent the overexpression or downregulation of pluripotency genes. For instance, inhibition of *Tcf3* (also known as *TCF711*) would increase the expression of *Oct4*, *Nanog*, and other pluripotency factors, thereby preventing exit from pluripotency [1–4]. Strikingly, triple deletion of *Etv5*, *Rbpj*, and *Tcf3* abolishes the differentiation ability of ESCs, thereby leaving them largely undifferentiated in the self-renewal circle, even in the presence of a differentiation stimuli [5].

Moreover, *p53* and *Jarid* induce the differentiation of pluripotent stem cells via inhibition of Nanog expression [6–8]. The nucleosome remodeling and histone deacetylase complex Mbd3-NuRD are decisive in the differentiation of mESCs [9]. In 2013, Austin Smith reported the role of Tsc1/2-Flcn-Tfe3 in pluripotency. The activation of the Tsc2-Flcn pathway would facilitate the transport of Tfe3 protein from the nucleus to the cytoplasm. Also, *Tfe3* is an upstream regulator of Esrrb. Therefore, the exit from the pluripotency network resulted from a cascade of amplification of various signals and controlled by specific programs [10, 11].

The genome-editing technology mediated by CRISPR-Cas9 was first developed for fundamental biology research in bacteria and archaebacteria [12–16]. Further, Jinek et al. designed a small guide RNA (sgRNA) by combining CRISPR-derived RNA (crRNA) and transactivating RNA (tracrRNA), well known as the CRISPR-Cas9 system, which help guide the Cas9 protein to digest DNA precisely [17]. A year later, Cong et al. first successfully performed genome directional editing both in human and animal cells, in which high mutation rates were obtained [18, 19]. The Cas9 protein could precisely identify targeted DNA sequences by the complementary base pairing of sgRNA and then perform double-strand DNA cleavage at a specific site [18, 20–23]. Recently, Zhang et al. first accomplished genome-wide CRISPR-Cas9 knockout (GeCKO) in human cells.

Additionally, genes involved in vemurafenib resistance were found in a melanoma model, including previously identified genes *NF1* and *MED12*, and the newly identified *NF2*, *CUL3*, *TADA2B*, and *TADA1*. At present, genome-wide CRISPR-Cas9 screening libraries have been adopted to screen for drug resistance, virulence, and tumor suppressor genes in multiple cell lineages and primary cells [24–33].

To search for the critical factors involved in the pluripotency exit of mESCs, herein, for the first time, we adopted the unbiased screening technique of genome-wide CRISPR-Cas9 knockout and successfully established the GeCKO-based model. We have identified genes that could facilitate the exit of mESCs from pluripotency. The new method has provided a paradigm and clues for further exploration of cell fate regulation.

## 2. Materials and Methods

### 2.1. Cell Culture

OG2 mESCs belong to the OG2-129 strain that was generated by crossing of CBA/CaJ×C57BL/6J mice (introduced from the Nanjing University National Genetic Engineering Mice Resource Library) and 129Sv/Jae mice (purchased from Beijing Charles River Company). mESCs were maintained, with feeder cells, in DMEM (high glucose, Hyclone) supplemented with 15% FBS (Gibco), 1× NEAA (Gibco), 1× GlutaMAX (Gibco), 1× sodium pyruvate (Gibco), 3 M CHIR99021 (MCE), 1 M PD0325901 (MCE), and 1000 units/ml LIF (Millipore). When the mESCs were cultured on 0.2% gelatin-coated culture dishes instead of feeder cells, mESCs were cultured in N2B27+2i/LIF medium containing DMEM (high glucose, Hyclone), 1× N2 (Gibco), 2× B27 (Gibco), 1× NEAA (Gibco), 1× GlutaMAX (Gibco), 1× sodium pyruvate (Gibco), 0.1 mM ME (Gibco), 3 M CHIR99021 (MCE), 1 M PD0325901 (MCE), and 1000 units/ml LIF (Millipore). Feeder cells were from ICR mice in Beijing Charles River Company. High-glucose DMEM (Hyclone), supplemented with 10% FBS (NTC), 1× NEAA (Gibco), and 1× GlutaMAX (Gibco) was used for feeder cell and 293 T cell cultures.

### 2.2. Differentiation of WT mESCs

Wild-type (WT) mESCs were cultured in N2B27+2i/LIF medium [10]. Cells were seeded onto a 6-well or 12-well plate at a density of 2 × 10^4^ (2E4), 4E4, 6E4, 8E4, and 10E4. The 2i/LIF was withdrawn after 12 hours or 24 hours of culture. The differentiation time points were 48 hours, 72 hours, and 96 hours. Subsequently, the cells were collected, and RNA was extracted for qPCR; the total cellular RNA was extracted by TRIzol reagents and then reverse transcribed into cDNA. RT-PCR (qPCR) was performed with the cDNA as a template. qPCR was performed using the primer sequences displayed in Table 1. The qPCR protocol is as follows: firstly, predeformation needs 93°C for 2 min, then amplify for 40 cycles: 93°C for 1 min, 55°C for 1 min, 72°C for 1 min, and finally 72°C for 7 min for extension.

### 2.3. GeCKO-sgRNA Library Preparation

Mouse genome-scale CRISPR knockout (GeCKO) v2.0 pooled library is the second edition of the genome-wide CRISPR gene knockout plasmid library, purchased from Addgene (#1000000053). Pooled lentiCRISPRv2 expression vectors containing the GeCKOv2 library were provided as two half-libraries A and B, at a concentration of 50 ng/*μ*l. It contains 130209 independent sgRNAs in total, which correspond to 20611 target genes (six sgRNAs/genes, with three of them in libraries A and B, respectively), 1175 target miRNAs (library A), and 1000 control sgRNAs (nontargeting, three in each of the A and B libraries). CRISPR libraries can be transformed into bacteria via electroporation for amplification following the manufacturer's instruction strictly [23, 24].

### 2.4. Lenti-Cas9-sgRNA Plasmid Construction

The candidate genes were selected based on preliminary screening results. For each gene, two sgRNAs that had been generated by positive enrichment and had relatively higher count numbers were selected from the GeCKO (v2.0) library. Specifically, two gRNAs (guide RNAs), 5′-TCAGCTCGTCGTTCGCTCCG-3′ and 5′-CCGAGCAGCGACAGCGCTT-3′ for targeting *Tcf7l1*; 5′-AACAGATCGTCCATGCAGTG-3′ and 5′-TGAGGGCTTACCATCACCAT-3′ for targeting *p53*; 5′-ACTAACATCGTTGCTAGTAG-3′ and 5′-CACAACTCCAGTGAAGATAG-3′ for targeting *Jarid2*; and 5′-CCGCTGCTGCCCTTGCCCAT-3′ and 5′-CATCTCCTTGCTTCCTAAAG-3′ for targeting *Fbxw7*, were cloned into the gRNA-expression plasmid lentiGuide-Puro.

The cohesive terminus sequence complemented with the end of the linearized lenti-Cas9 vector was added to one end of the sense/antisense sequence. Further, it was attached to the linearized vector after annealing. Following transformation, plate coating, bacterial colony picking, and amplifying, the plasmid could be used for transfection if the sequencing results were correct.

### 2.5. Plko-shRNA Plasmid Construction

pLKO.1 is a replication-incompetent lentiviral vector chosen by the TRC for expression of shRNAs [34]. The shRNA oligos for pLKO.1 are provided free by the website (https://www.sigmaaldrich.com/china-mainland.html). First, the shRNA sense sequence of the target gene was obtained, and the antisense sequence was constructed based on the sense sequence. The skeleton of forwarding oligo is 5′CCGG-(x)-CTCGAG-(y)-(T)TTTTT(G)3′, while the skeleton of the reverse oligo is 5′AATT(C)AAAAA(A)-(x)-CTCGAG-(y)3′. Of those, x and y represent the specific palindrome sequence of shRNA of interest, respectively. After annealing, the synthesized primer was ligated into the linearized pLKO.1 vector (digestion with EcoRI and AgeI). Following transformation, plate coating, bacterial colony picking, and amplifying, the plasmid could be used for transfection if the sequencing results were correct.

### 2.6. Lentivirus Production

Both half-libraries (A and B) were used, and viral particles were produced independently from A and B before being used to infect recipient cells. 5 × 10^6^ HEK293T cells were plated for each 10 cm dish and cotransfected the next day with 12.5 *μ*g library A or B plasmids (lentiCRISPRv2), 5 *μ*g pMD.2G, and 7.5 *μ*g psPAX2 lentiviral packaging plasmids, using 2.5 ml of transfection solution which contained 156.25 *μ*l of 2 M CaCl_2_, 1250 *μ*l of 2 × HBS, and 1068.75 *μ*l H_2_O. PRlenti-mCherry was used as the transfection control. Cells were incubated 10-16 hours, and then media was replaced with fresh culture medium. After 48 hours, viral supernatants were collected and passed through a 0.45 *μ*m filter (Merck Millipore) in the presence of 8 *μ*g/ml polybrene (Sigma-Aldrich) and then used to infect recipient cells.

### 2.7. Construction of the Cas9-Blast High-Expressed mESC Lineage

The lentivirus was packaged with lentiCas9-Blast plasmid, and used for wild-type mESC infection. The culture medium was changed after 8 hours of infection, and cells were further cultured for 48 hours. Then, Cas9-expressing cells were selected with 2 *μ*g/ml blasticidin for 3 days. In order to get the high expression of Cas9 cell lines, those Cas9-expressing mESCs were seeded at an extremely low density, ensuring that each clone originated from a single cell. 3 days later, about 20 clones were chosen for expansion and establishment into the Cas9-expressing cell lines. The cell lines with the highest expression level of Cas9 mRNA were used for further infection with GeCKO-sgRNA library virus.

### 2.8. GeCKOv2 Screening Genes Which Contribute to mES Exiting from Naïve Pluripotency

Cas9-expressing mES cells were transduced with the GeCKO v2 library. Briefly, 2.4 × 10^7^ Cas9-expressing mESCs per well were plated into a 15 cm plate in mES culture media supplemented with 2i. And cells were infected with the lentiviral library at the same time. The mESCs were infected at a low MOI (0.3) to ensure that most cells receive only 1 viral construct. After 8 hours infection, the culture medium was changed to fresh mES culture media supplemented with 2i. Then, after 48 hours of culture, transduced Cas9-expressing mES cells with the GeCKOv.2 library were treated with 1 *μ*g/ml puromycin (Thermo Fisher Scientific) for another 4 days. In order to compare initial abundance when plasmid libraries were first transfected into cells and evolution screening abundance in passage, cells were collected after 48 hours of puromycin selection as input 1 and input 2 were collected after additional 3 passages (9 days) culture in mES media with 2i.

For screening genes involved in the pluripotency exit in mES with GeCKOv2 library, transduced mESCs were further selected in the medium of 2i/LIF withdrawal for 2 passages, then collected as the experimental group control. At this step, most of cells were differentiated and could not self-renew. These selected cells were cultured again in the mES medium with 2i for one passage and most of the differentiated cells could not survive since they could not adapt to the ground-state mES medium. We collected those cells which could be rescued, as the experimental group.

### 2.9. Genome Extraction and PCR Resequencing

Genomic DNA was extracted from these cells using isopropanol-extracted method and sgRNA sequences isolated by PCR. All samples were digested with nuclear lysis buffer containing protease K till clear and colorless. Then, the RNase and protein precipitations solutions were added in sequence to remove RNA and protein contamination. After precipitation with isopropanol, the sample was rinsed with newly prepared 70% ethyl alcohol and air-dried. The sample was dissolved in water; sequencing was performed.

Library construction with next-generation sequencing: the library was constructed with a two-step method. As reported previously [24], first, the PCR fragments containing the library were amplified from input 1, input 2, and the experimental group. Primers used to amplify lentiCRISPRv2 sgRNAs for the first PCR are sense, 5′-AATGGACTATCATATGCTTACCGTAACTTGAAAGTATTTCG-3′ and antisense, 5′-CTTTAGTTTGTATGTCTGTTGCTATTATGTCTACTATTCTTTCC-3′. A second PCR reaction was carried out on the resulting amplicons to add adapter sequences for the sequencing system and barcodes to discriminate each sample after multiplex polymerase chain reaction. Primers for the second PCR include both a variable length sequence to increase library complexity and an 8 bp barcode for multiplexing of different biological samples. Primer message can refer to Zhang et al. [24]. Amplification was carried out with 18 cycles for the first PCR and 24 cycles for the second PCR. After the second PCR, resulting amplicons were purified using Agencourt AMPure XP beads (Beckman Coulter) and mixed and sequenced in a NextSeq500 (Illumina).

### 2.10. MAGeCK Analyses and GO Analyses

The next-generation sequencing data were processed with the MAGeCK algorithm [35]. By comparing input 2 and input 1, significantly decreased genes were obtained, and GO analyses were conducted.

### 2.11. Statistical Analyses

The data were presented as mean ± SD. The SPSS22.0 and GraphPad Prism software and model-based analysis of genome-wide CRISPR-Cas9 knockout (MAGeCK) were adopted for statistical analyses and plotting. Also, unpaired Student's *t*-test was used for comparison between two groups. *P* < 0.05 was considered significant.

## 3. Results

### 3.1. The GeCKO-Based Screening Strategy for Key Factors of mESCs in the Exit from Pluripotency

**Figure 1:**
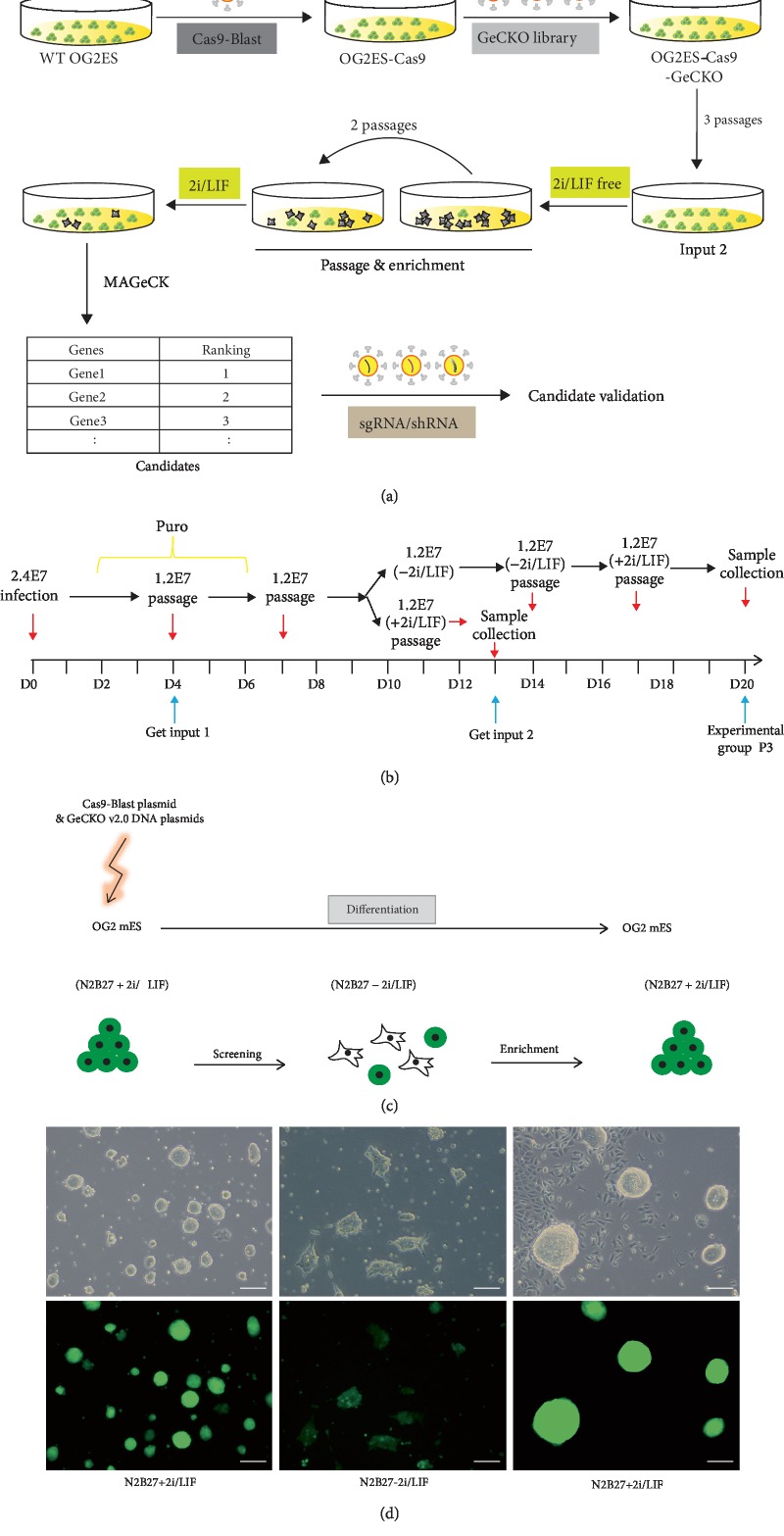
The screening procedure: (a) outline of the research procedure and (b) technical route. 2.4 × 10^7^ lentiCas9-Blast-expressing mESCs were transfected with GeCKO (v2) lentiGuide-Puro in 2i/LIF, and then input 1 was collected after 48 hours puromycin selection and input 2 after additional 3 passages (9 days) cultured in N2B27+2i/LIF; experimental group was collected after 2 passages of mutation period and 2 passages of 2i/LIF withdrawal and one more passage in 2i/LIF conditions. 2.4E7 stands for 2.4 × 10^7^ mES cells. (c) OG2 mESCs were transfected with Cas9-Blast plasmid and GeCKO v2.0 DNA plasmids in N2B27+2i/LIF medium. Differentiation was enabled by inhibitor removal and enrichment performed by restoring 2i/LIF. (d) Phase and fluorescence images of mES cells at the three stages. Scale bar, 100 *μ*m.

GeCKO (v2) lentiGuide-Puro plasmid library virus infected into the mESC high-expressing lentiCas9-Blast at a low multiplicity of infection (MOI = 0.3). Cells were induced into differentiation for two passages in the pluripotency exit medium (N2B27-2i/LIF) for screening. Then, pluripotency maintenance medium (N2B27+2i/LIF) was added for one passage (enrichment) [24]. Subsequently, the sample was collected, and genomic DNA was extracted for next-generation sequencing. MAGeCK algorithm was adopted for data management, thereby evaluating the effectiveness of this model. Furthermore, candidate genes were screened (analyses of the screening data). With two rounds of experiments, we could identify the target gene (Figure 1(a)).

The screening process includes the following five steps (Figures 1(b)–1(d)). (1) Virus packaging: the 293 T cells were transfected with lentiGuide-Puro, psPAX2, and pMD.2G at a ratio of 5 : 3 : 2. After 48 hours of culture, the culture media containing the virus were collected, and directly used for cell transfection, or stored at -80°C, 10 ml/tube. (2) Transduction: the mESCs expressing high levels of lentiCas9-Blast were seeded onto eight 15 cm plates, with 2.4 × 10^7^ cells per plate. Then, the cells were transduced with a virus; the tilter of which was precalculated. After 8 hours of transfection, the culture medium was changed to the mESC culture medium containing FBS and 2i/LIF. After 48 hours of transduction, the cells were treated with 1 mg/ml puromycin for 2 days. The cells that have been successfully transduced and expressed sgRNA were screened and collected. The DNA was extracted and defined as input 1. (3) Mutation: the cells containing lentiGuide-Puro sgRNA library were cultured in the N2B27+2i/LIF medium for 3 passages, and the extracted DNA was defined as input 2. (4) Screening: the 2i/LIF was withdrawn from the culture medium, and the cells were further cultured in the N2B27 medium without 2i/LIF for 2 passages. Most of the cells differentiated, could not self-renew, and easily died, but the cells with target sgRNA did not differentiate and survived. (5) Enrichment: the 2i/LIF was readded into the N2B27 medium. Then, the viable differentiated cells would die because they fail to adapt to the ground-state mESC culture medium. However, those undifferentiated cells or those able to recover would proliferate significantly and accumulate. These cells were collected and their DNA was extracted and defined as the experiment group.

### 3.2. Establishment of the Screening Model and Technical Route

**Figure 2:**
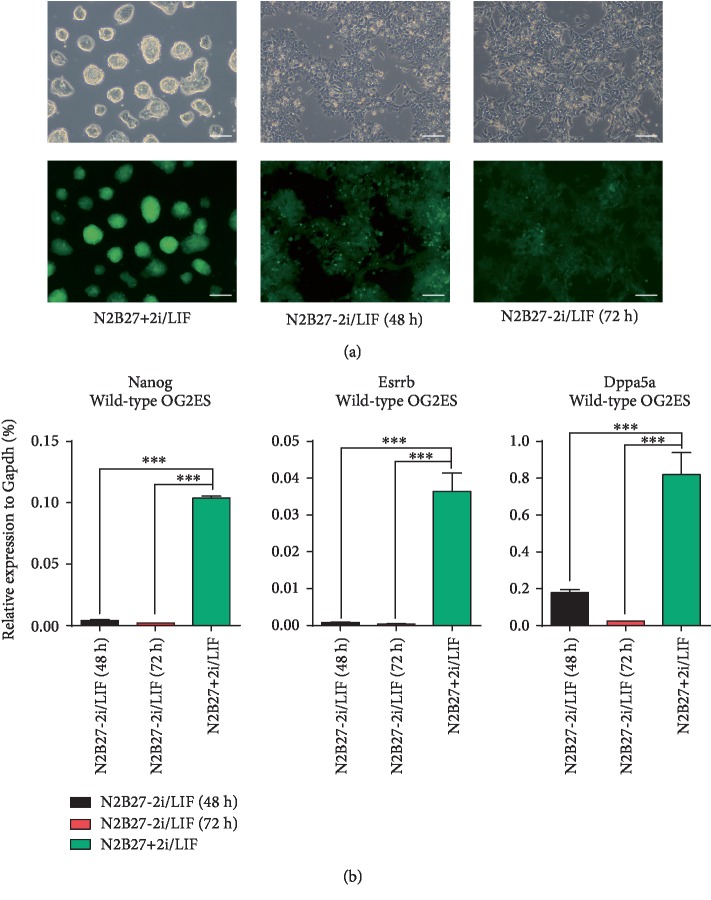
OG2 mESCs treated by withdrawing 2i/LIF. (a) All groups were cultured in N2B27+2i/LIF for the first 24 hours, and then differentiation was enabled by inhibitor removal for another 48 or 72 hours in the experimental groups, while the control group was cultured in N2B27+2i/LIF for additional 48 hours. Scale bar, 100 *μ*m. (b) RT-qPCR analysis of the expression of three pluripotency factors in wild type. OG2 mESCs treated by withdrawing 2i/LIF. The error bar represents standard error, ^∗∗∗^*P* < 0.001 vs. the control group, *n* = 2.

Following multiple experiments on the WT mESC differentiation, we have established the following differential conditions: The mESCs were subcultured in 12-well plates at a density of 80,000 cells/well and refed with a normal ground-state multipotency maintenance medium (N2B27+2i/LIF). In the experimental group, the culture medium was replaced with the multipotency exit medium (N2B27) without 2i/LIF (N2B27-2i/LIF) after 24 hours of culture. After induction of differentiation for another 48 and 72 hours, cells were collected for further experiments. In the control group, cells were stilled cultured in the N2B27+2i/LIF medium for another 48 hours before they were collected. Then, we extracted RNA from each culture, and the expression of pluripotent factors was determined by RT-qPCR. We found that (1) as for the time point of culture medium replacement, it would be better if we change the culture medium 24 hours after adhesion, rather than 12 hours. The advantages are mainly better cell attachment and cellular morphology; (2) as for the differentiation time, the decline of three pluripotent factors, namely the *Nanog*, *Esrrb* and *Dppa5a*, was ideal; and (3) as the differentiation time period is prolonged, the number of dead cells increases while viable cells decreases. To sum up, when conducting genome-wide gene knockout, we would differentiate the cells during 3 passages, we induce each passage for 72 hours, thereby enhancing screening efforts and reducing false positive. However, when screening validation was conducted later, the induction differentiation period of each passage was 48 hours because there were fewer candidate genes and thus more accurate. Meanwhile, it would shorten the experimental cycle, reduce cost, and alleviate the impact of additional factors. The results are shown in Figure 2.

### 3.3. GeCKO Screening Data Analysis

Input 1 represents the ground state where the GeCKO (v2.0) lentiGuide-Puro sgRNA plasmid library was just transfected into the cells, but no effects were observed. As the reference of input 2, it reflects the initial abundance when the plasmid library is first transfected into cells. Accordingly, input 2 reflects the evolutionary screening during standard passages, when the GeCKO (v2.0) lentiGuide-Puro sgRNA plasmid library affects all cells.

**Figure 3:**
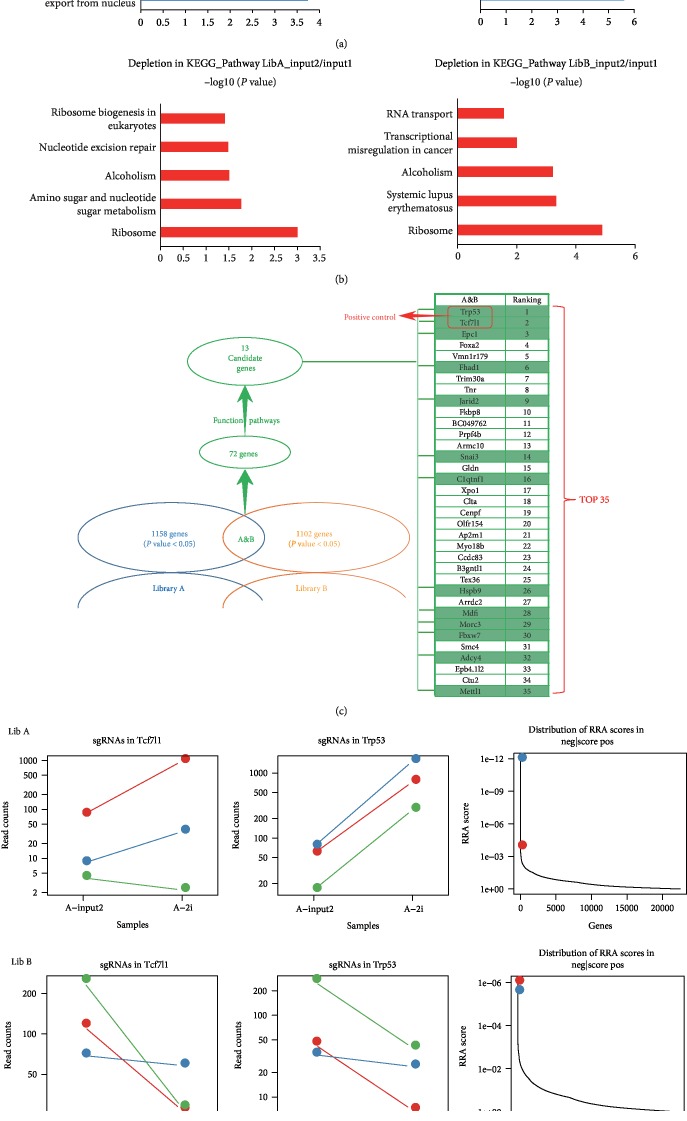
Screening data GO analysis. (a, b) Comparison of input 1 with input 2 for genome-scale negative selection screening. The most significantly depleted genes in mESCs transfected with both GeCKO (v2.0) library A and B. (c, d) GeCKO screen in mESCs reveals genes whose loss confers commitment resistance. The most significantly enriched genes including two positive controls *Trp53* and *Tcf7l1* (ranking first and second), as well as another 70 candidate genes (A/B-2i/LIF: E-group with library A/B).

GO analyses showed that in the comparison of input 2 with input 1, the category or cellular localization of the most depletion of sgRNAs targeting genes are as follows: ribosomal large subunit biogenesis and ribosomal large subunit export from the nucleus, ribosome biogenesis, ribosomal small subunit export from the nucleus, translation, and phospholipid transport (Figure 3(a)). Accordingly, the functional categories of those most depletion of sgRNAs targeting genes in KEGG pathway are as follows: ribosome biogenesis in eukaryotes, nucleotide excision repair, alcoholism, an amino sugar, and nucleotide sugar metabolism, and ribosome (Figure 3(b)). The above observation indicates that the essential survival genes were depleted during routine passage (input 2/input 1). That is, cells would gradually die during a routine passage when the genes that keep the cells survival are knocked-out. As compared with the primary library with average diversity, it would reveal a significant reduction in the diversity of sgRNAs in the surviving cells. This above observation of adverse selection has verified the efficacy of our model and indicates that our screen was effective in achieving knockout of endogenous gene targets.

Knockdown of *Tcf7l1* and *p53* is included as a reference. By comparing E-group with input 2, we found enrichment of multiple siRNAs that target the positive control genes (*Tcf7l1* and *p53*) with an extremely high MAGeCK RRA score (robustrRank aggregation, RRA) in library A and B (Figure 3(d)). The MAGeCK analyses results were ranked by the comprehensive enrichment in these two libraries, and the top two were *p53* and *Tcf7l1*, with a total of 72 genes screened (*P* ≤ 0.05). Based on the full consideration of the known function, signaling pathway, and the relevant epigenetic regulation effect of each gene, 13 candidate genes (including the positive control genes, *Tcf7l1* and *p53*) were selected from the top 72 genes (Figure 3(c)) for further experimental verification.

### 3.4. Differentiation Verification after the Lenti-Cas9-sgRNA Knockout of 13 Candidate Genes

**Figure 4:**
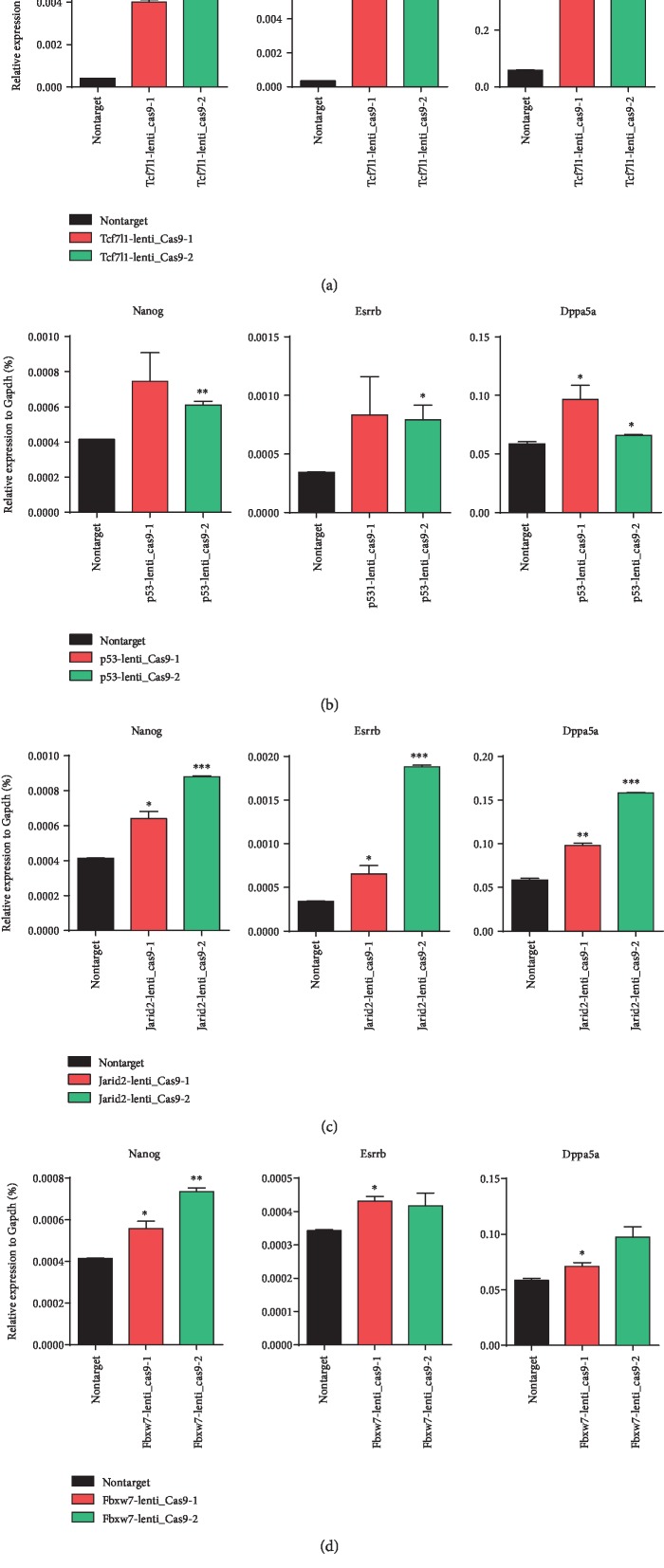
RT-qPCR analysis of the three pluripotency factors' expression in wild-type OG2 mESCs transfected with lenti-Cas9-sgRNA of (a) Tcf7l1, (b) p53, (c) Jarid2, and (d) Fbxw7 and treated by withdrawing 2i/LIF. The error bar represents standard error, ∗*P* < 0.05,^∗∗^*P* < 0.01, ∗∗∗*P* < 0.001 vs. Nontarget.

For the 13 candidate genes, two apparently enriched sgRNAs were selected from the GeCKO v2.0 plasmid library for each gene and cloned into the pRlenti-Cas9-puro lentiviral vector. The Cas9 and sgRNA were integrated into the mESCs by virus transfection and infection. 2i/LIF was removed after routine culture for one passage. After 48 hours of differentiation, RNA was extracted for determination of the mRNA expression levels of three pluripotency factors: *Nanog*, *Esrrb*, and *Dppa5a*. Of those, *Nanog* and *Esrrb* are core pluripotency factors. The higher the expression level, the better the pluripotent state. As shown in Figure 4, with Nontarget being the control, the knockout of *Tcf7l1*, *p53*, *Jarid2*, and *Fbxw7* simultaneously showed significant effects on the transcriptional repair of the three pluripotency factor genes, *Nanog*, *Esrrb*, and *Dppa5a*, which encode pluripotent factors. Of those, the *Nanog* transcription level was the most important. The third round of screening was performed on the above four genes.

### 3.5. Differentiation Verification after the Plko-shRNA Knockdown of the Four Genes Screened

**Figure 5:**
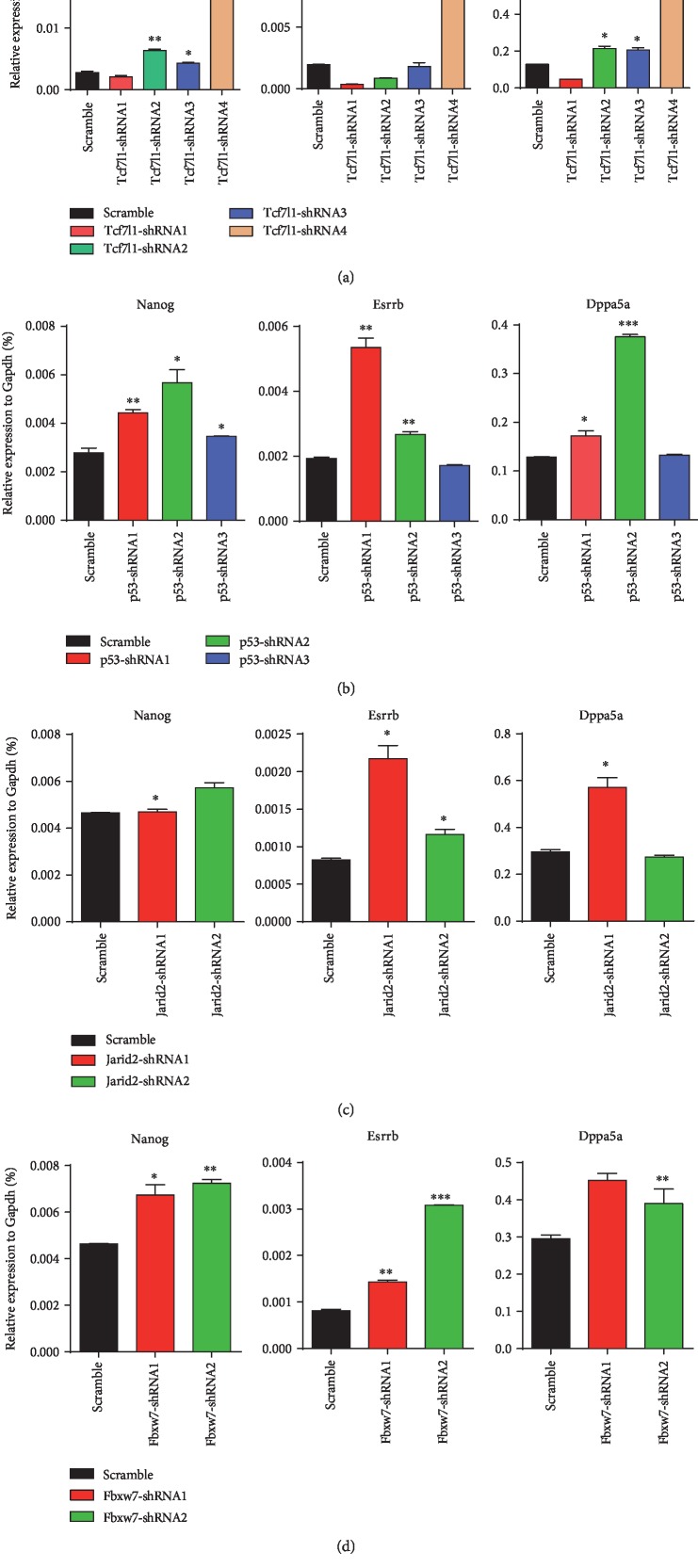
RT-qPCR analysis of the expression of three pluripotency factors in wild-type OG2 mESCs transfected with plko-shRNA of (a) *Tcf7l1*, (b) *p53*, (c) *Jarid2*, and (d) *Fbxw7* and treated by withdrawing 2i/LIF. The error bar represents standard error, ^∗^*P* < 0.05, ^∗∗^*P* < 0.01, ^∗∗∗^*P* < 0.001 vs. Scramble.

**Figure 6:**
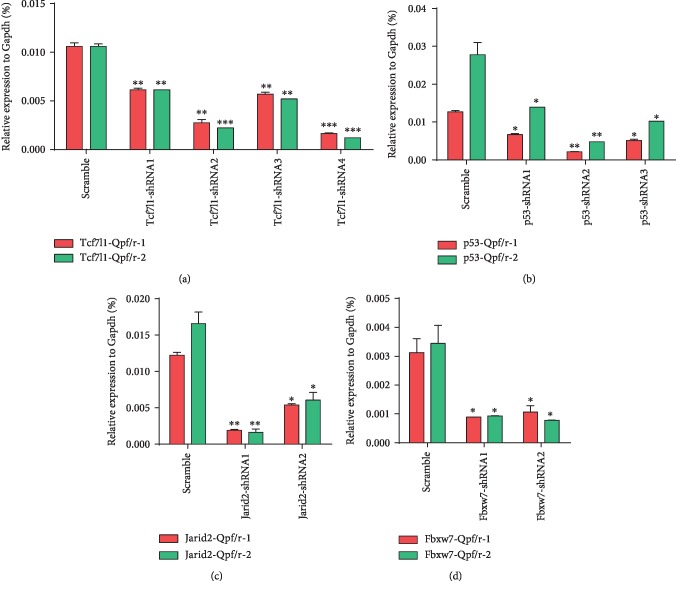
The knockdown efficiency of shRNAs targeting the four candidates: (a) *Tcf7l1*, (b) *p53*, (c) *Jarid2*, and (d) *Fbxw7.* The error bar represents standard error, ^∗^*P* < 0.05, ^∗∗^*P* < 0.01, ^∗∗∗^*P* < 0.001 vs. Scramble.

By RT-qPCR (Figures 5 and 6), the knockdown effect of shRNAs targeting the two reference genes could match with the corresponding cellular pluripotency state (2-4 shRNAs per gene), which is significantly different from the control group (Scramble). That is, multiple shRNA targeting of the target genes generates better gene knockdown effect as well as higher transcription levels of the pluripotency factors. The results indicated that reduced transcript of the target genes would enable ESCs to withstand differentiation conditions and impair commitment. In the two rounds of screening, the knockdown of *Jarid2* and *Fbxw7* was well consistent with the maintenance of pluripotency, suggesting that these genes were involved in the pluripotency exit of pluripotent stem cells.

## 4. Discussion

Large-scale genetic screening holds great value in studies of molecular biology, cytobiology, oncology, genetics, and pathology [36, 37]. With reverse genetics, we have a chance to identify key genes and signaling pathways that affect cell fate transition, disease occurrence, and development. Nevertheless, the characteristics of low throughput, time consuming, and effort demanding limit the wide application of whole-genome screening. Otherwise, we could only inhibit the target gene at the posttranscriptional level, rather than complete knockout. We first adopted the CRISPR/Cas9 genome-wide gene knockout technique in the study on the pluripotency exit of pluripotent cells. Genes were targeted by six sgRNAs, and different sites were simultaneously edited. By complete knockout of 20611 target genes and 1175 target miRNAs, we successfully apply genome-wide screening for the key genes involved in the exit from pluripotency. Compared with siRNA library screening, genome-wide gene knockout screening based on CRISPR/Cas9 has the advantages of stable knockout phenotype, little dose effect, and long duration effect. Also, it diminishes the effect of loss of expression, as compared with shRNA.

The key design in our study is screening strategy. The principle of screening strategy is to provide a hostile environment. On this condition, the viable cells may contain more candidate genes [38–40]. 2i and LIF are of great importance in ground-state pluripotency maintenance. Pluripotent cells cultured in the medium without 2i and LIF would differentiate or die, and cell death would increase with the passage number. However, if gene knockout could completely or partially substitute for 2i and LIF, postpone the differentiation of pluripotent cells and even maintain cellular pluripotency, it may delay or even prevent the exit from pluripotency, indicating a critical role of the gene in the exit from pluripotency. We successfully established the screening model with that phenotype. Moreover, high-throughput sequencing was adopted to identify the candidate genes, and all data obtained at one time. The results were further validated with triplicates, and the target gene was identified.

Our screening process depends on both abundance and transfection efficiency. The cells were transfected with low-titer library viruses, enabling a good abundance slightly after transfection and avoiding sgRNA preference before the screening. Second, to ensure sufficient cell numbers and phenotype as well as to reduce the false positive and false negative rates as much as possible, we enhanced screening efforts by increasing the passage number in a differential medium and conducting repeated screenings. To rule out the interference of other factors on stem cell pluripotency and reduce the variability of our results, the experiment was repeated, applying strictly the same optimal protocol every time.

The CRISPR/Cas9 gene knockout-based screening is a more thorough inactivation of genes than screening using RNAi knockdown and is not affected by the dose-effect of gene expression caused by the heterogeneous RNAi knockdown efficiency. In addition, because it is a gene knockout, screening may also be more sensitive. What's more, the effect of RNAi is short term. With the cell culture, the knockdown efficiency of RNAi will gradually decrease, which will bring uncontrollable effects. On the contrary, the gene knockout cells will have the stable inheritance for a long time [10, 41]. Leeb and his colleagues used the transposition system to screen in haploid cells [42]. The disadvantage is that the insertion of the transposon system in the genome is biased, and the protein coding sequence only accounts for about 2% of the genome. Therefore, most of the insertion does not effectively achieve the purpose of gene knockout. In comparison, Cas9-based screening can cause mutations at relatively precise locations in the genome. At the same time, insertions within gene bodies are likely to produce protein mutations with new functions. In addition, haploid cells are not normal cells after all, so the screening results can only be used as a reference, and still need to be verified in normal diploid cells.

The work of MacDougall and colleagues utilized the strategy of direct differentiation to EpiLC [43]. Fgf2 is also added after 2iL is removed. The screening results may also be affected by Fgf2, and our method is a kind of random differentiation after the removal of 2i/LIF from the mESC maintenance medium. The work of Li and Villegas used Rex1-GFP reporter to screen cell clones resistant to differentiation [44, 45]. Remarkably, these screening results based on that reporter system both seem to be related to the mTOR signaling pathway. In contrast, we screened for the clones resistant to differentiation by whether mESCs can restore stem cell-like self-renewal capabilities and can be maintained in the N2B27+2i/LIF medium after the process of differentiation.

## 5. Conclusion

We have identified 4 genes that promote the exit from pluripotency, namely the *TCF7l1*, *p53*, *Jarid2*, and *Fbxw7*. These have already been identified as positive genes [1–7, 46]. The fact that we identified these genes with large-scale genome screening technique has proved the effectiveness of our method. We successfully combined two hot topics in the field of life and science, namely the stem cell fate regulation and gene editing technology. In this way, we not only provide a new method for tackling specific scientific problems like the exit from pluripotency but also establish a mode of thinking for life and science research. Taken together, our studies could provide new and significant insights for further researches in the domain of stem cell.

## Figures and Tables

**Table 1:** Details of primers used for the qRT-PCR analysis of selected genes used in the PCR experiments.

Gene name	Primer sequences	Annealing T (°C)	GenBank accession number
*Gapdh*	F: 5′-AACTTTGGCATTGTGGAAGGGCTCA-3′ R: 5′-TTGGCAGCACCAGTGGATGCAGGGA-3′	60	NM_001289726.1

*Nanog*	F: 5′-CTCAAGTCCTGAGGCTGACA-3′ R: 5′-TGAAACCTGTCCTTGAGTGC-3′	60	NM_028016.3

*Esrrb*	F: 5′-TTTCTGGAACCCATGGAGAG-3′ R: 5′-AGCCAGCACCTCCTTCTACA-3′	60	NM_011934.4

*Dppa5a*	F: 5′-GAAGTATTCCAGGTCCAGTC-3′ R: 5′-TTGAGAGCCGTAAATGAAGA-3′	60	NM_025274.3

## Data Availability

The data used to support the findings of this study are included within the article.
